# Consumer Perceptions of Botanical Sources of Nutrients: A UK-Based Visual Focus Group Study Exploring Perceptions of Nettles (*Urtica dioica*) as a Sustainable Food Source

**DOI:** 10.3390/foods14213702

**Published:** 2025-10-29

**Authors:** Eleanor Bryant, Danni Walters, Chloe Mellor, Louise Neilson, Natalie Rouse, Alina Warren-Walker, Amanda J. Lloyd, Robert J. Nash, Tennessee Randall, Laura L. Wilkinson

**Affiliations:** 1 School of Psychology, Faculty of Medicine, Health and Life Science, Swansea University, Swansea SA2 8PP, UK; eleanor.bryant@swansea.ac.uk (E.B.); 953811@swansea.ac.uk (C.M.); 2356500@swansea.ac.uk (N.R.); 2 School of Psychology, Faculty of Social Sciences, University of East Anglia, Norwich NR4 7TJ, UK; danni.walters@uea.ac.uk; 3 Twisted Orange, 14 Ballard Grove Sidford, Sidmouth EX10 9EP, UK; louise@twisted-orange.co.uk; 4 Cleobury PM, Canolfan Bwyd Trimsaran, Trimsaran SA17 4AA, UK; ali@cleoburypm.com.uk (A.W.-W.); mandy@cleoburypm.com (A.J.L.); 5 PhytoQuest Ltd., Plas Gogerddan, Aberystwyth SY23 3EB, UK; robert.nash@phytoquest.co.uk

**Keywords:** nettles, sustainable food consumption, visual focus group, plant-based, botanical, UK consumers, perceptions

## Abstract

Increasingly, attention is being paid to the underutilised wild edible nettle plant (*Urtica dioica*) as a healthy and sustainable food source. However, little is known about UK consumers’ acceptance of nettles and supplements containing nettles. This study explored UK consumers’ perceptions of nettles as a food source and nettle-based powder supplements, using visual focus group methodology (i.e., creative drawing tasks and group discussion). A total of *n* = 34 participated in the study, with each participant engaging in one of five visual focus groups. Barriers to consumption and how consumers envisaged using nettle-based supplements were also explored. Inductive thematic analysis was used to analyse group discussions and pictures were analysed using visual content analysis drawing on the principles of content analysis, semiotics, and iconography. Findings revealed limited awareness amongst consumers about nettles as a food source, with sensory properties and prior experiences negatively affecting perceptions of nettle consumption. Concerns over processing and the inclusion of carrier ingredients reduced consumer trust in a nettle powder supplement viewing these as ‘ultra-processed’ and unhealthy. A preference for natural additional ingredients was revealed, potentially signalling an opportunity to engage and educate consumers around sustainable eating. Transparency in labelling information may improve consumer confidence and trust in nettle powder supplements.

## 1. Introduction

Climate change and food production systems are intrinsically connected; food production systems are a key driver of climate change but are equally susceptible to the effects of climate variation [1]. Estimates suggest that global food production systems contribute 34% to global greenhouse gas (GHG) emissions, primarily owing to agricultural and land use practices [1,2]. Equally, current food systems fail to optimise global human health [3]. In Europe, growing attention is being paid to developing policy guidance for improving sustainable food production systems, yet sustainable food consumption (SFC) remains overlooked at policy level despite its inclusion in the United Nations (UN) Sustainability Development Goal (SDG) 12 ‘responsible production and consumption’ [4]. Arguably, however, country-wide policy interventions (e.g., via meat pricing increases) may not be best placed to support a dietary transition towards the increased consumption of sustainable foods [5]. By contrast, consumer-focused approaches (e.g., increased availability of alternative plant-based foods, educational interventions) which account for social and environmental contributors to dietary patterns and preferences may provide a more effective approach [5].

Importantly, however, SFC lacks an agreed definition and often narrowly focuses on environmental and agricultural practices whilst neglecting important socio-economic and cultural factors [6]. Indeed, a recent literature review identified common themes which suited a broader definition of SFC encompassing these four factors: health (e.g., diet is sufficient in vitamins and minerals and they provide adequate proportions of macronutrients), socioeconomics (e.g., foods are available and affordable), sociocultural (e.g., pleasure, future generations), and environment (e.g., soil health, biodiversity, carbon emissions) [4]. Specifically, guidelines indicate that healthy and sustainable diets should be rich in wholegrains, legumes, fruit, and vegetables, only including moderate amounts of dairy, eggs, and small amounts of red meat [7]. Whilst increased consumption of sustainable foods has the potential to improve global health and lower GHG emissions, it requires a dietary transition which depends on consumer willingness and acceptance [8,9].

Food innovation and technology will likely play a crucial role in the transition to a sustainable food system which delivers environmental benefits alongside supporting global health and nutrition in the face of population growth [10,11]. However, perceived negative health effects over food processing in innovative foods are a consumer concern [12]. Indeed, early research showed that consumer interest in foods processed with novel technologies (e.g., genetic modification) was lower when consumers associated such technologies with increased health risks [13]. Processing remains a consumer concern, with consumers linking higher levels of processing to perceptions of unhealthiness [14]. The NOVA classification system is currently used to categorise foods according to four levels of processing (from unprocessed/minimally processed to “ultra processed”); although this system is criticised for lacking a scientific foundation, potentially leading to consumer confusion [15]. Furthermore, technologies which serve to preserve and improve the functional properties of sustainable foods often require additional ingredients (e.g., Clear Gum, a modified starch), which may be classified as ultra-processed, further eroding consumer confidence and trust in novel products [16]. In contrast, foods which are perceived as natural—ideally grown using traditional and natural methods—are preferred by consumers and are more likely to be viewed as healthy [14,17]. Consequently, food innovators are increasingly exploring foods which have historical and traditional uses to develop novel sustainable products which have consumer appeal, but a greater understanding of the barriers to consuming such products is needed.

Botanical sources of nutrition, such as the common and fast-growing stinging nettle, are gaining attention as a healthy and sustainable food [18,19]. Native to Europe, Asia, Africa, and North America, the perennial stinging nettle or *Urtica dioica* L. is a member of the Urticaceae family of herbaceous plants and is a wild edible plant [18]. Nettles grow well in temperate climates, albeit more prolifically in nitrogen-rich soils [20]. Nettles are also ecologically beneficial as they improve biodiversity and their presence acts as a sign of good soil health [19]. Nettles have a range of uses in agriculture and other industries. They are fibrous, so are potentially well suited to the textile industry and provide a natural form of fertiliser and pest control [21,22,23]. Additionally, they require low inputs and make good use of marginalised land, so they are considered ideal for organic farming systems [21]. Specific to human health and nutrition, nettles also have a wide range of medicinal benefits which come from the leaves, roots, stems, and seeds [20]. Historically, nettles have been used to alleviate symptoms associated with arthritis, rheumatic conditions, and protect against cardiovascular disease [18,19,20,24]. They can also help with allergies, owing to their purported antihistamine properties, so everyday use such as drinking nettle tea, or as a home remedy, is considered beneficial to reducing hay fever symptoms [19]. Despite these traditional medicinal uses, nettles have no authorised health or medicinal claims according to the European Food Standards Agency (EFSA) and currently remain on the ‘botanical on-hold’ (i.e., claims under review) register [25]. Despite the wide range of uses alongside reported ecological and health benefits of nettles, they remain underutilised as a source of nutrition [26] and underrecognised from a regulatory perspective.

As a food source, nettles are highly nutritious and contain bioactive compounds which are important to health [19]. These include phenolic compounds, carotenoids, lignans, polysaccharides, flavonoids, and sterols [18]. Nettles are a natural source of vitamins (e.g., vitamin C, vitamin A), minerals (e.g., iron, calcium, magnesium, and potassium), and antioxidants which come from polyphenols (phenolics, flavonoids, and carotenoids) [18,20,24]. Nettles are a valued food crop across many countries and can help with food security in times of food shortages and drought [27]. In Nepal, nettles have high consumer value and are commonly used as a vegetable [19]. In contrast, an ethnographic study in Ethiopia found that nettles hold less value because they are linked to a person’s economic context so those on lower incomes are more likely to gather and consume nettles [27]. Nettles are traditionally used within teas and recipes (e.g., soups, pesto, curries) or added as an ingredient to supplement foods such as bread and beers [20,28]. Consumer trends show a rapid growth in plant-derived dietary supplements in recent years [29,30]. These are made using several different drying methods (e.g., spray drying, freeze drying) which are used to process botanicals and create a stable product whilst preserving health benefits [31,32]. One study which surveyed consumers across six European countries showed that a wide range of botanicals are used [33]. However, the consumption of supplements containing nettles was substantially lower than those containing ginkgo biloba and evening primrose. A similar finding is reported elsewhere [34], whereby echinacea and ginkgo biloba were most commonly used. Even so, there is a growing interest in nettle-based products, owing to their nutritional profile [18]. For example, chemical analysis of a nettle powder composed of dried, ground nettle leaves showed a superior nutritional profile and higher protein content when compared to barley and wheat flours [35]. Despite a history of traditional usage in many countries and strong consumer interest in botanicals, little is known about UK consumers’ perceptions of nettles and whether products containing nettles are acceptable or desirable to consumers.

Prior research using focus group methodology as an approach to explore the acceptance of novel and underutilised sustainable foods (e.g., algae), encouragingly showed that consumers were open to trying novel food sources, recognising the dual benefit to human health and the environment [36]. Consequently, focus groups may advantageously elicit deep insights into beliefs and perceived barriers to consuming sustainable foods [6]. This methodological approach may be of relevance to the exploration of consumer perceptions of nettles as a source of nutrition considering potential safety concerns and perceived barriers, owing to their unique sensory characteristics [27]. However, a less commonly used focus group approach, but one which is viewed as having benefits over traditional discussion-based methods, is the visual focus group [37]. Visual focus groups use participatory art-based creative tasks which are inclusive in nature because they are suited to more diverse participant groups and may overcome language and communication barriers [37]. As an approach, visual focus groups support participants to reflect more deeply on their beliefs and experiences and elicit less cliché and ‘out of the box’ responses [37,38]. Participatory approaches may help identify the barriers and solutions to support a dietary transition towards SFC [39]. Thus, visual focus groups may be particularly useful in exploring consumer perceptions of novel or underutilised foods and perceived barriers to their consumption and processing.

There is an increased interest in nettles as a healthy and sustainable food source. Like algae, although it has historic and traditional usage, nettles have become less used, suggesting that consumers in the UK may be unfamiliar with their consumption. Research exploring the desirability and acceptance of novel foods containing nettles is still needed. Therefore, the aim of this study was to explore UK consumers’ perceptions of nettles and nettle-based soluble, powdered supplements as a botanical source of nutrition using a series of visual focus groups. The creative art task and focus group discussions explored participants’ perceptions of nettles as a general food source, an exploration of participants’ preferences (e.g., format, uses) for supplements containing nettles, and how they may incorporate, specifically, a nettle powder supplement into their diet. A further aim of this study was to explore perceived barriers and challenges towards consuming nettles and nettle-based powder supplements.

## 2. Materials and Methods

### 2.1. Participants

Five visual focus groups were conducted during November and December 2024, falling within an acceptable range (4–8 groups) to achieve data saturation [40]. The number of participants attending focus groups ranged from 6 to 8 participants. Participants were recruited to a study advertised as ‘Understanding the acceptance of a plant-based food supplement through art—a visual focus group study’ using the following methods (1): Swansea University-based adverts (e.g., School of Psychology participant pool, staff user groups, and email distributions) for four campus based focus groups, and (2) via social media platforms (e.g., via Facebook) and face-to-face events in a local community hub for one community-based focus group. Most participants received a £15 voucher as compensation for their time, although some eligible participants (i.e., psychology students) opted for School of Psychology participant pool credits. Participants were eligible to participate if they were over 18 and did not self-report a current or historical diagnosis of an eating disorder. In total, 38 participants were recruited from either Swansea University (*n* = 31, 81.6%) or the wider Swansea community (*n* = 7, 18.4%); however, *n* = 3 participants were unable to attend their focus group and *n* = 1 withdrew, leaving *n* = 34 participants who completed the study. Participants age ranged from 18 to 85 years old (M = 34.09, SD = 16.92). Further participant characteristics are presented in Table 1.

### 2.2. Materials

The aim of the visual focus group was to use individual and collective drawing tasks to explore perceptions of nettles as a botanical source of nutrients. The research materials and visual focus group structure were as follows:

#### 2.2.1. Screening Questionnaire

A basic screening questionnaire was hosted on the online platform Qualtrics (www.qualtrics.com; accessed between 20 November 2024 and 4 December 2024) and surveyed participants’ age, gender, and ethnicity.

#### 2.2.2. Visual Focus Groups

To meet the current aims, a novel protocol was developed, drawing on the visual focus group concept by [37], the theory of planned behaviour (TPB; previously identified as a suitable framework for exploring SFC [41,42,43]), and from prior learnings from pilot work considering sustainable eating more generally, by a subset of the research team. The TPB was used to shape the research protocol for the drawing tasks and subsequent focus group discussions. For all drawing tasks, participants were provided with suitable art materials (A4 and A3 paper, coloured and grey lead pencils, and felt tip art pens). The first drawing was devised to explore participants’ attitudes and norms towards consuming nettles. The session began with an individual drawing task (Task 1) whereby participants were asked what consuming nettles meant to them, followed by a researcher-led group discussion which explored the participants’ artwork. After Task 1, researchers provided an explanation to participants on the health benefits of nettle consumption as a food supplement in spray-dried powder form and the spray-drying processes involved in creating powdered supplements. This included information about the role of additional (i.e., carrier) ingredients to preserve bioactive ingredients and improve the quality and solubility of the finished powder. Following this, participants were divided into small groups (maximum of four participants) aligning to the view that small groups are needed for art-based focus groups [37]. The second drawing task (Task 2) was devised to explore perceived behavioural control (PBC) and behavioural intentions (BI) towards consuming a nettle-based powder supplement. Participants were asked to collectively draw as a group (1) when they might intentionally include nettles for consumption mapping onto BI, and (2) what they considered as the main barriers or challenges to nettle use in supplement/ingredient form mapping onto PBC. Task 2 was similarly followed by a researcher-led group discussion.

### 2.3. Procedure

Ethical approval for this study was granted by Swansea University’s School of Psychology Study Ethics Committee (Project Code: 13798). Participants who responded to study advertisements were directed to a sign-up form to indicate their preferred focus group and gather contact information. Once focus groups were organised, participants were emailed the visual focus group arrangements (e.g., date and time of session, location details), a participant information sheet, and a link to Qualtrics (www.qualtrics.com; accessed between 20 November 2024 and 4 December 2024), which contained the consent form and requested basic demographic information. The information sheet outlined that the purpose of the study was to explore an art-based approach focusing on the acceptance of sustainable food supplements involving collective art-based tasks and group discussion. At this point, participants were not informed of the study’s focus on nettles, to avoid a self-selecting sample. Data protection and confidentiality arrangements were also provided.

All visual focus groups lasted one hour and were led by two researchers (TR and CM) to ensure consistency across sessions and followed the protocol outlined above (see Section 2.2.2. Visual Focus Group). Art-based tasks and group discussions were led by the researchers and audio recorded for data analysis purposes. Afterwards, participants were thanked for their time and provided with a debrief form with the researchers’ contact details. The form explained that the study’s primary objective was to explore consumer acceptability of the everyday use of nettles, alongside providing information about the health benefits of nettle consumption.

### 2.4. Data Analysis

The following approach was adopted for data analysis to develop themes based on the drawing tasks and accompanying discussions which took place during and after the drawing tasks. The researchers recognised the symbiotic relationship between group discussions and the participants’ drawings [44,45,46]. Although the discussions and images were initially analysed separately, they were subsequently considered together to enhance interpretation. First, inductive thematic analysis [47] was applied to the data across a series of phases. Phase one involved transcription of each focus group by one researcher (DW), followed by data familiarisation. In phase two, data were systematically coded across transcripts starting with one transcript. Recurring codes and new codes were identified across subsequent transcripts, and before the end of the coding process no new codes were identified, indicating data saturation. This was followed by a review process where codes were revisited to ensure coherence across the dataset. In phase three, codes were grouped into initial themes and then checked by a second researcher (TR). Both researchers agreed to the need for further refinement to ensure themes were distinct. In phase four, themes were refined through an iterative process and grouped into broader themes and sub-themes, reviewing these alongside the data to ensure accuracy. In phase five, themes were defined by both researchers (DW and TR) and given titles to ensure themes were distinct and were represented by supporting quotes. Second, image analysis was conducted using a new approach to visual analysis, combining the principles of content analysis (descriptive), semiotics, and iconography (connotative) [48,49]. A combination of these approaches was used as some drawings were easily interpretable, such as icons of cooking and nature, whereas others required referencing to the accompanying discussion for deeper contextual interpretation. DW first reviewed all drawings, making initial observations. Each picture was analysed and coded according to visual content, with distinct objects and symbols (e.g., a person, mug or cross) counted as discrete elements. Elements were then grouped into preliminary categories to capture recurring visual features and themes. Themes from the image analysis closely aligned with those generated through thematic analysis though this was not predetermined. Salient visual elements and interpretations were integrated with the thematic analysis to enrich and support overall theme generation, thus providing richer insights into the data.

## 3. Results

Consumer perceptions of nettles as a food source are summarised across the following three major themes (see Table 2).

### 3.1. Theme 1: “Why Would You Want to Eat a Cactus?” “It’s Healthy for You”: Familiarity with Consuming Nettles

Participants showed varying levels of familiarity with nettle consumption and their reported health benefits. Some participants had no familiarity with nettle consumption as a source of nutrition, whereas other participants showed awareness of the traditional uses of nettles for health but equally showed little desire to consume them. Here, past painful experiences of being stung by nettles played an influential role on the perceived desirability for nettle consumption. For those who had consumed nettles previously, participants cited potential health benefits and novelty as underlying motivational factors. Only a few participants reported continuing nettle consumption. Thus, participants’ experiences of nettles and familiarity with nettle consumption can be clustered into the following three sub-themes.

#### 3.1.1. Sub-Theme 1: Never Heard of Eating Nettles

**Figure 1:**
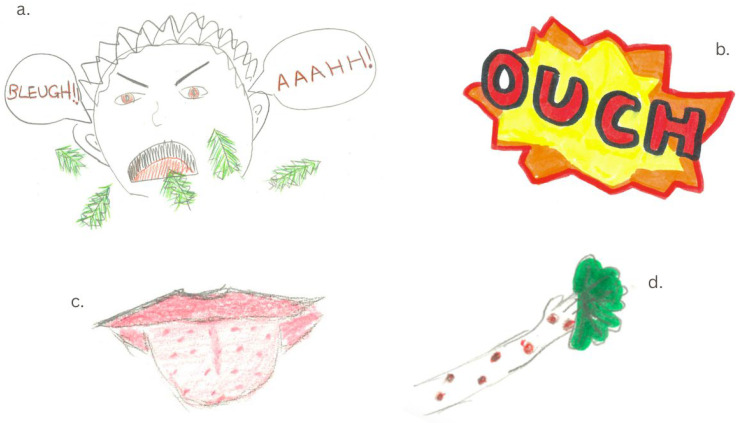
Participant drawings representing experiences and expectations about nettle consumption. (**a**) person in pain after eating nettles with two speech bubbles exclaiming disgust and pain; (**b**) red and yellow abstract spikey speech bubble symbol with the word “OUCH”; (**c**) mouth with a tongue covered in red stinging marks from nettles; (**d**) arm covered in red stinging marks from nettle holding a leaf.

Those who had never heard of eating nettles reacted with confusion or disgust towards the idea of consuming nettles. As depicted in Figure 1, participants tended to draw on their past experiences of having been stung by nettles which subsequently informed their expectations about the texture and safety of consuming nettles (see Figure 1a–d). These experiences and expectations provided a justification to participants as to why nettles should be avoided as a food source, particularly owing to their stinging properties.

“*There’s a horrible texture that you would associate with it. […] Yeah, definitely. Because that’s the whole thing is the texture of it. Because it like gives you bumps and makes you itch and things.*”

Participants drew arms and tongues that were sore from stinging nettles (see Figure 1b,d), as well as sad and pained faces of people who had eaten raw nettle (see Figure 1a). Bright red and orange were used in these drawings, emphasising the concern and danger associated with participants’ experiences of being stung by nettles (Figure 1b).

#### 3.1.2. Sub-Theme 2: Heard of Eating Nettles but Not Tried Them

Some participants were aware that nettles could be consumed and had traditional uses for health, but this had not resulted in increased motivation for participants to seek them out as a source of nutrition.

“*My nan recommended drinking it one time […] and I passed […] it was around spring when I was getting hay fever bad, so she was like, “This will help,” and I was like, “I’ll just suffer.*”

Some participants continued to cite concerns around the safety of eating stinging nettles, whilst others simply had not tried them but had knowledge of their reported health benefits.

“*I think it’s more like, experience with nettles is they’re painful. So then the idea of something, they’re like,* “*Drink this painful drink.*” *It’s like,* “*Danger juice.*”

**Figure 2:**
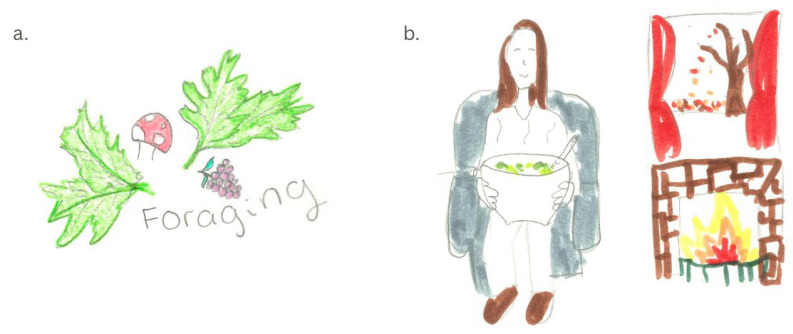
Participant drawings associating nettles with nature, warmth and cosiness. (**a**) green leaves, a red mushroom and berries representing foraging; (**b**) a happy person sat in an armchair holding a hot bowl of nettle soup beside a fireplace and autumn scenery out the window.

Although levels of familiarity with nettles varied, those who held a positive view of nettle consumption depicted nettles as ‘cosy’ and comforting in their drawings often with images of nettle tea and soup, and scenes from nature (see Figure 2a,b). For example, Figure 2b shows a happy person who is sat inside during autumn beside a fire with a hot bowl of soup.

“*I’m thinking it’s warm and cosy […] I only knew about soup and that last year.*”

#### 3.1.3. Sub-Theme 3: Tried Eating Nettles

**Figure 3:**
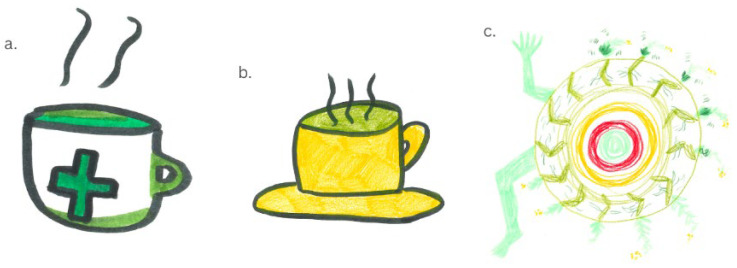
Participant drawings representing perceived benefits of nettle consumption. (**a**) a green medicine cross on a mug of warm nettle tea; (**b**) a yellow mug of warm nettle tea; (**c**) green and red abstract representation of the positive energising benefits of nettlesParticipants held mixed views about continued use of nettles, with few expressing a desire to continue drinking the tea they had bought or consuming nettles in meals after their initial experience. There were a variety of reasons for this, such as disliking the flavour, novelty experience, and the accessibility of nettles. However, some experiences and perceptions were positive towards nettle consumption and supported continued use.

For those who had consumed nettle before, they typically cited traditional formats such as soup and tea. These were particularly prominent in the drawings, with participants drawing hot bowls of green soup and hot tea (see Figure 3a,b). The perceived health benefits and novelty of nettles were reported by participants as the primary reasons for consuming them. Knowledge of the benefits of the tea were drawn using the common green medicine cross on a mug of tea (see Figure 3a) or through abstract representations that depicted the energy they felt after consuming it as described by one participant (Figure 3c).

“*I liked it. I enjoy it. I think it has that kind of earthy maybe tones and definitely not sweet. And I enjoy all those kinds of stuff, especially if they are taken from a field or something. I come from Poland, so maybe like we’re like taking all different kind of like flowers and leaves and stuff, drying them out and taking them. And I enjoy things like that myself.*”

### 3.2. Theme 2: “Disguise It in Your Own Way” “Done and Dusted”: Sensory Attributes and Convenience Influences Format

The second drawing task explored perceptions towards the use of a novel nettle powder supplement and views over different supplement formats. Participants had preferences for a range of supplement formats, often citing alternative formats such as flakes, seasoning, and capsules as well as the proposed powder supplement presented to them. Participants’ enthusiasm for a nettle-based supplement varied, whereby perceptions of flavour, control over use, and versatility were viewed as important factors which would drive consumer acceptability and subsequent use.

Perceptions about how the supplement powder could be incorporated into foods and beverages as a food ingredient were motivated by participants’ assumptions about flavour and whether it was perceived as sweet, savoury, or flavourless. Participants initially showed a preference for a powder supplement which would be suitable for smoothies but expanded on their vision suggesting incorporating the supplement into the savoury foods they cook everyday such as soups, mash, gravy, and pasta sauces. This was largely motivated by the desire to disguise presumed negative tastes as well as making it easy to combine into their regular meals.

“*[…] I think the, like, benefits of it make me think of perhaps wanting to cook it into comfort food as well. So, you know, the things that you really love, and then adding something as well, where you know it’s not just the psychological benefits that you’re eating this meal, it makes you feel good. You know that you can have all these, like, dose of vitamins as well.*”

**Figure 4:**
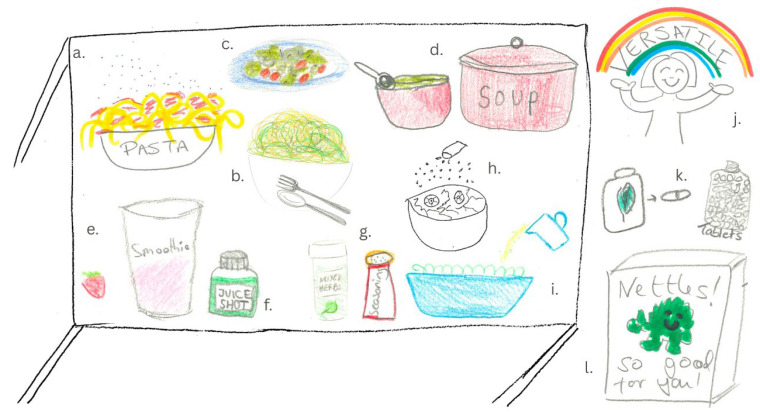
Participant drawings representing Theme 2. Table of different meals and uses of nettle powder showing the desire for versatility of the nettle supplement. (**a**) spaghetti with nettle supplement being sprinkled on top; (**b**) nettle pasta with a spoon and fork; (**c**) salad with nettles; (**d**) a red saucepan labelled with soup and a bowl of green nettle soup with a spoon; (**e**) a glass labelled smoothie with a strawberry suggesting the nettle powder is in the smoothie; (**f**) a green juice shot with nettle inside; (**g**) nettle supplement in mixed herb and seasoning shakers; (**h**) a bowl of salad being sprinkled with nettle seasoning; (**i**) a bowl of salad with a nettle vinaigrette being poured over it; (**j**) a happy person with a rainbow and the word ‘VERSATILE’; (**k**) depictions of nettle supplement in the form of tablets; (**l**) a box of nettle supplement with a green character and the words “Nettles! So good for you!”.

Several groups opted to draw a dining table with multiple different dishes (see Figure 4). This domestic setting and variety of meal types emphasised participant sentiment of being in control of using the supplement in their home-cooked food in a way that is versatile and easy to incorporate. There were a few drawings of smoothies and blenders (see Figure 4e); ultimately however, savoury meals were dominant across the drawings (Figure 4a–d). This reflected the dynamic of the discussion moving from smoothies to more diverse uses of a supplement.

Participants who perceived that the supplement flavour would be mild or neutral (i.e., flavourless) moved away from the idea of a powder supplement, preferring flakes as a format to enhance the nutritional value of foods. This was reflected in participants’ drawings as nettle seasoning being sprinkled over pasta and potatoes or even a nettle vinaigrette being poured over a salad (Figure 4g–i).

“*I said that we could add it to salads. That’s how I would personally use it as long as it was tasteless and it didn’t affect the salad. In the form of a tea, if it could be like shaken up and then put it into a tea or something like that. Or added to like a soup that’s already your favourite and it wasn’t going to add anything except for nutritional value to it.*”

“*If you were to sprinkle it on something you’d imagine it wouldn’t necessarily taste of much.*”

Participants who expressed concerns about the proposed powder supplement taste (i.e., having a bad flavour) and texture (i.e., affecting the consistency of foods) preferred for the supplement to come in the form of a capsule or gummy. A capsule was also seen as a positive in regard to ease of consumption of the supplement and was therefore viewed as a convenient way to provide the health and nutritional benefits of nettles.

“*I think a capsule could be beneficial for people who are busy or don’t have time to incorporate it in their cooking or anything like that. It’s easy to take. It would be done and dusted without having to do anything else.*”

“*Like having it in a vitamin form or a gummy because then you just have it, you don’t have to worry about the taste or how much extra in […] Like making it in that form or you buying it in that form? […] Buying in that form, yeah. And then its already, you don’t have to worry about how much you use. It’s already done for you, you just take it as a multi-vitamin.*”

### 3.3. Theme 3: “I Can’t See Why Anyone Wouldn’t Take It” “There Are Nicer Things to Drink” It Might Be Healthy but…

**Figure 5:**
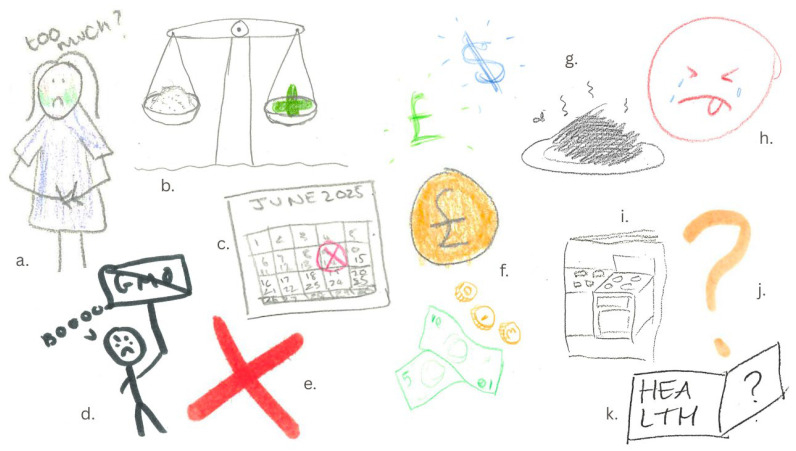
Participant drawings representing Theme 3. Depictions of an array of different concerns participants had about nettle supplements. (**a**) a person clutching their stomach with green cheeks and the words “too much?”; (**b**) a weighing scales with nettle powder in one side weighed up with an green medicine symbol on the other; (**c**) a calendar depicting concerns around shelf-life; (**d**) an angry person holding a protest sign with the word “GMO” crossed out; (**e**) a red cross; (**f**) a series of different visuals of money; (**g**) a pile of smelly black food that had been ruined by the nettle powder; (**h**) a sad crying face with the tongue stuck out; (**i**) a cookbook; (**j**) an orange question mark; (**k**) a book with the word “HEALTH” on one page and a question mark on the other.

The second drawing task further explored participants’ perceptions of the barriers and challenges to consuming nettle-based supplements. Participants noted that one of the main barriers to overcome was the negative perceptions associated with nettles that may prevent consumers from considering a nettle-based product in the first place. This sentiment of concern is emphasised in the sad, crying face with its tongue sticking out in disgust (Figure 5h).

“*[…] convincing people that it would actually be good, because most of our, initial reactions was—[…] negativity in general, or pain or disgust. So, that would be a big issue, it’s just general perceptions.*”

#### 3.3.1. Sub-Theme One: … It Will Not Be Nice to Eat

These negative perceptions were primarily driven by the continued negative assumptions around the nettle-based supplement’s sensory attributes, which was believed to negatively impact on consumer appeal. A drawing of a pile of black food that smelled (Figure 5g) emphasised participant concerns that the powder could ruin a meal due to its influence on consistency, taste, and appearance. Participants also discussed choosing alternative health supplements with comparable health benefits to avoid the need for consuming nettles.

“*And there are so many other [options], like, they say about the health benefits, but there are nicer things to drink to get it, so why would I go for something that has bad connotations?*”

For some participants, however, the potential health benefits associated with nettles counteracted the perceived barriers around the organoleptic characteristics.

“*I don’t care if it tastes bad, if I really need it.*”

#### 3.3.2. Sub-Theme Two: … It Is Ultra-Processed Which Reduces Its Healthiness

Participants were informed of the use of carrier ingredients (to preserve bioactive compounds and powder quality), and three examples were used (maltodextrin, potato starch, and gum arabic). This led to concerns amongst some participants regarding the processed and unnatural sound of some of the proposed carrier ingredients and presumed negative effects on the healthiness of the supplement, reducing product appeal. Drawings of a person protesting GMO and large red crosses were prominently used to depict perceptions about the unnatural and industrial processing of the supplement with carriers in (Figure 5d,e). As a result, potato starch was preferred as a natural-sounding and familiar ingredient.

“*Well, I know about potatoes.*”

“*And I was just thinking about how much of the potato starch that you have in that, versus the actual nettles. So, it is just like 5 percentage of the nettle and then 95 or something else. Why would I have the actual pill, you know, I might as well just have the leaf?*”

Even so, participants varied in how much of a barrier they perceived the carrier ingredients to be, with some expressing that it would prevent them consuming a nettle supplement whilst others admitted they paid little attention to such ingredients.

“*They can’t be that horrid or awful if they’ve been used in other things already.*”

#### 3.3.3. Sub-Theme Three: … It Is Too Expensive and Difficult to Find

Affordability and accessibility were important factors for participants for a nettle powder supplement. 

For pricing, affordability was important and furthermore, as nettles grow locally in the UK, participants believed that the price should reflect this. Icons of money featured prominently across all group drawings highlighting it as a key barrier to purchase (Figure 5f).

“*I’d like to think it would be reasonably priced just because it is easy to grow and harvest nettles.*”

Others cited the overwhelming feeling of going into health food stores. If the product was not affordable or accessible, participants would turn to alternative products for the same health benefits.

“*And then if it was accessible, like if it was in easily to access in [global brand] or it was in your general supermarket rather than you having to go to [UK health food shop brand name] and like fish through all the other supplements that it was just there on the shelf as a powder that you can spread on your salad and on the package it said it helps with arthritis say for example. And then people would be like right I’ll try that and see if it helps.*“

#### 3.3.4. Sub-Theme Four… I Do Not Know How Much to Consume

Many concerns were also raised about consuming the right dosage of the supplement and saying that clear instructions would be needed on how best to use the supplement to retain its benefits. These concerns were emphasised in a drawing of a person with green cheeks clutching their stomach suggesting they are ill from consuming too much of the supplement (Figure 5a). As well as through weighing scales where there is a medicine cross on the one side depicting the health benefits and the pile of supplement on the other (Figure 5b). This concern also prompted discussions surrounding the format of the supplement coming in a capsule which would help control the dose that you were taking. The shelf life of the supplement was also a concern (Figure 5c), which a capsule format could potentially avoid.

“*The thing about powder, I would think, when you’re talking about the negative about the powder that you can now use, it’s going to be a minimal amount, when it’s dry too. So, it’s getting the right, correct measurement. […] Whereas in the capsule you don’t have to worry about the amount.*”

#### 3.3.5. Sub-Theme Five… I Do Not Trust the Health Claims

Scepticism surrounding the trustworthiness of purported health claims of the supplement was raised as a barrier to purchasing and consuming a nettle supplement. Participants further cited a reluctance to purchase a product to avoid trying something novel whilst others indicated it would be important to verify health claims by looking at research supporting the use of a nettle powder supplement.

“*But I also concern if it’s in powder form. Would it affect the effectiveness of the ingredient to my body? So, I would like, I think I would just read the studies and see what kind of, what in what form that the, I don’t know if it’s a trial or something that the participants take during that trial and then see if that’s powder. (Participant 2: Yeah.) And then that’s, I’ll know it’s effective because they’re using the same thing I’m using.*”

Participants drew question marks and books on health to emphasise their desire to know more about the supplement before purchasing (Figure 5j,k). Clear packaging and labelling about the health benefits were viewed by some as a way of overcoming these barriers. One participant even drew a cookbook showing an openness to learning how to incorporate the supplement into their foods but demonstrating that they would not be confident to do so without knowing how (Figure 5i).

Participants were informed about some of the health benefits that nettles could provide, particularly in relation to arthritis. Whilst this was viewed as a good health benefit, it was felt that it may lack relevance and appeal to all consumers compared to broader health messages suggesting that how information about the powder supplement is presented to consumers is important to perceptions of desirability.

“*If you want to market it, it would have to be, as broad as possible, if that makes sense? Because, you know, if it just said, benefits arthritis, then I feel like I wouldn’t be too keen on it. Or my people, my age demographic wouldn’t be too… into that. But if it was like, antioxidant, has these vitamins, it does this and this it would be a bit more enticing I suppose?*”

## 4. Discussion

Extending previous research exploring UK consumers’ perceptions of sustainable foods with a history of traditional use (e.g., [36]), this study aimed to explore consumers’ perceptions of nettles, an underutilised yet nutritious food source, and preferences for food supplements and ingredients containing nettles using visual focus group methodology. Using this participatory approach, this study further aimed to identify the perceived barriers and challenges to consuming nettles and nettle-based supplements. A combined data analytic approach was used which included inductive thematic analysis [47], content analysis [49], semiotics, and iconography [48] which resulted in the emergence of three overarching themes. Familiarity with nettles as a source of food varied across participants. In their natural form, negative attitudes towards nettle consumption were shaped by painful past experiences and limited knowledge of their health benefits. However, increased understanding of the health benefits of nettles led to more favourable attitudes towards nettle consumption. In powdered form, attitudes were more positive towards introducing nettles into diets. Furthermore, participants held greater perceived intentions to use the powder, consequently outlining the variety of ways a powder could be incorporated into diets. Intentions to use a powdered supplement were influenced by different factors, including attitudes, negative perceptions of sensory attributes, concern over ‘ultra-processed’ carrier ingredients, and uncertainty about the trustworthiness of health claims. Intentions were also affected by PBC, whereby lack of accessibility and affordability were viewed as barriers.

Traditionally, nettles have been consumed as wild vegetables, often added as an ingredient in soups, curries, and pasta [35,50]. Similarly, participants in this study who previously had consumed nettles tended to describe traditional uses such as preparing nettles in teas and soups. The norms for consuming nettles were centred on cultural and generational aspects, which were discussed by participants as important drivers as to why they had consumed nettles. Historically across Europe, foraging for wild edible foods was commonplace and comprised an important part of a person’s diet, but requires knowledge to be passed on between generations and a connection to the local landscape [51]. In recent years, however, social, economic, and ecological changes have disrupted this process, leading to a loss of knowledge transfer alongside plant loss owing to changes to land use, local habitats, and ecology [51,52]. In some regions across Europe, traditional knowledge of wild foods and a connection to the land is still present. For example, one study of people living in rural areas on the Polish, Lithuanian, and Belarusian borders, showed that traditional knowledge and use of wild plants, including nettles, was good, even for plants that had disappeared from the landscape [52]. Here, cultural connections to food through social traditions supported the retention of knowledge, suggesting that cultural relevance is important for underutilised or even extinct wild plants that require knowledge exchange [52].

However, whilst participants in our study were familiar with the nettle plant, many were unfamiliar with the notion of nettles as a source of food and nutrition. Nettles have a range of nutrition and health benefits, e.g., [18,19,20], grow abundantly in the UK [53], and have a history of traditional use [28] but socio-economic and land use changes [51] may have weakened this knowledge transfer between generations resulting in low recognition of nettles as a valuable and sustainable food source. This may prove a key challenge for food innovators, owing to consumer avoidance or rejection of novel or unfamiliar foods [54,55]. In contrast to research showing an openness to consuming novel sustainable foods [36], some participants in this study reacted with disgust and expressed concern about the safety of nettle consumption, associating nettles with danger and pain. Poor recognition of nettles as a food source alongside awareness and experience of nettles as a plant with stinging properties may prove an additional barrier to food novelty which needs consideration when assessing consumers’ acceptance and desire for nettles and nettle-based supplements. Although further research is warranted, this may signal a need for consumer education or intervention to address such barriers.

Modern lifestyles and changes to dietary habits have led to the demand for healthy foods which are convenient [56]. Processing nettles into a powder supplement using spray-drying methods with carrier ingredients provides consumers with convenience whilst preserving the health and nutritional benefits of the nettle plant. Carriers are essential ingredients which support the quality (e.g., texture, solubility, and shelf life) of powdered food supplements [57]. Perceptions of processing directly impact consumer decision making and purchasing intentions. Indeed, research has found that convenient ready-to-go foods designed to support health and nutrition negatively affected consumer choices when they were described as ‘ultra-processed’ [58,59]. Similarly, findings here revealed concerns about inclusion of ‘ultra-processed’ carrier ingredients, potentially eroding trust in the health benefits of nettles as a powder supplement. In contrast, natural carriers (i.e., potato starch) were viewed as healthier ingredients, in line with previous research which shows that consumers show strong preferences for products which are perceived as natural because they are viewed as healthier [17,56]. Clear labelling of healthy and sustainable foods may help to overcome some of these barriers [60] and was also identified by participants as a way to promote the health benefits of nettles whilst alleviating consumer concerns. Future research could examine consumers’ preferences for the labelling and messaging used within nettle-based food supplements.

### Limitations

First, this study aimed to recruit participants from community settings in addition to university-based participants, although only one focus group was carried out in a community setting. Additionally, limited information about the participants’ characteristics was collected and whilst information about some demographic information (e.g., age, gender) was collected, it was not linked to focus group data, restricting the exploration of differences in perceptions across groups. In the future, conducting more focus groups in community settings and gathering further information about participants’ characteristics would be beneficial. This would help achieve greater diversity and representativeness in sampling and allow for a deeper understanding of factors which may influence consumers’ perceptions, supporting the generalizability of findings. Second, this study focused on exploring consumer perceptions of nettles; however, other underutilised botanicals with good nutritional profiles (including dandelions and wild garlic [61,62]) warrant attention in respect to consumer acceptance of sustainable foods. Finally, creative and participative approaches have been previously used to explore consumers’ adoption of sustainable diets, with researchers reflecting that individual tasks may be more effective in drawing out individual perspectives [39], aligning to researchers’ observations in this study. Thus, future research in this area should consider a refined methodological approach.

## 5. Conclusions

This study shows that consumer acceptance of nettles and nettle-based supplements is influenced by prior experiences with the nettle plant and perceptions of its sensory properties. Whilst there is growing demand for foods that are healthy, sustainable, and convenient, concerns about the processing of additional ingredients and perceptions of unnaturalness may erode consumer trust, creating barriers for consumers during product selection. A challenge for food innovators is therefore improving consumer confidence and trust in health supplements including those containing nettles. Production transparency in how nettle-based powder supplements are processed alongside clear labelling information may serve to alleviate some of these challenges.

## Figures and Tables

**Table 1:** Participant characteristics.

Participant Characteristics		*n* (%)
Gender	Man	7 (20.6%)
	Woman	25 (73.5%)
	Non-binary/third gender	2 (5.9%)
Ethnicity	White	27 (79.4%)
	Asian	6 (17.6%)
	Prefer not to answer	1 (2.9%)

**Table 2:** Theme map showing the themes, sub-themes, and supporting quotes.

Themes	Sub-Themes	Supporting Quotes from Focus Groups
Theme 1. “Why would you want to eat a cactus?” “it’s healthy for you”: Familiarity with consuming nettles	Never heard of eating nettles	“I just thought about it as like, ‘why would you want to eat a cactus?’”
	Heard of eating nettles but not tried them	“It’s meant to have positive things, but I can’t imagine doing it because to me it’s pain.”
	Tried eating nettles	“I think I can remember from when I was a child, so my grandma used to put stinging nettles in soup.”
Theme 2. “Disguise it in your own way” “done and dusted”: Sensory Attributes and Convenience Influences Format.		“You can add it to something and disguise it in your own way.”
Theme 3. “I can’t see why anyone wouldn’t take it” “there are nicer things to drink” It might be healthy but…	… it will not be nice to eat.	“The taste is something that would put me off trying/buying it.”
	… it is ultra-processed which reduces its healthiness.	“Because you don’t want it to be like a natural supplement and then there’s like highly processed ingredients that, that defeats the point.”
	… it is too expensive and difficult to find.	“So if it’s fairly affordable, I’ll use it, I’ll add it to my food, health benefits are good, but if it’s probably over three quid, for the size of not gravy granules, garlic granules you buy, I’d say if it’s over three quid for that, I wouldn’t get it, just because it’s like why would I be buying that when I could go eat kale or something?”
	… I do not know how much to consume.	“How much nettle do you need to consume to get all those benefits? […] You know, like is it a dash of powder and suddenly you’re cured, or is it like a whole like five pounds?”
	… I do not trust the health claims.	“We’re worried who would be doing it. Who would be making it and for what reasons. If it was a commercial manufacturer who thought wow this is a great idea free, virtually free, nettles and we can sell it as a super super food.”

## Data Availability

The data presented in this study are available on request from the corresponding author due to privacy.

## Abbreviations

The following abbreviations are used in this manuscript:

GHG	Green House Gas
SFC	Sustainable Food Consumption
SDG	Sustainable Development Goals
UN	United Nations
TPB	Theory of Planned Behaviour
EFSA	European Food Standards Agency
